# Supplementary material for: Genomic Analysis of the Natural Variation of Fatty Acid Composition in Seed Oils of Camelina sativa
Source: Biology (Basel). 2025 Sep 5;14(9):1199. doi: 10.3390/biology14091199 (PMC12467081; doi:10.3390/biology14091199)
Supplement: Supplementary file 1 [file biology-14-01199-s001.zip › biology-3834074-supplementary.pdf]

Supplementary Materials

Genomic Analysis of Natural Variation of Fatty Acid Composition in Seed Oils of *Camelina sativa*

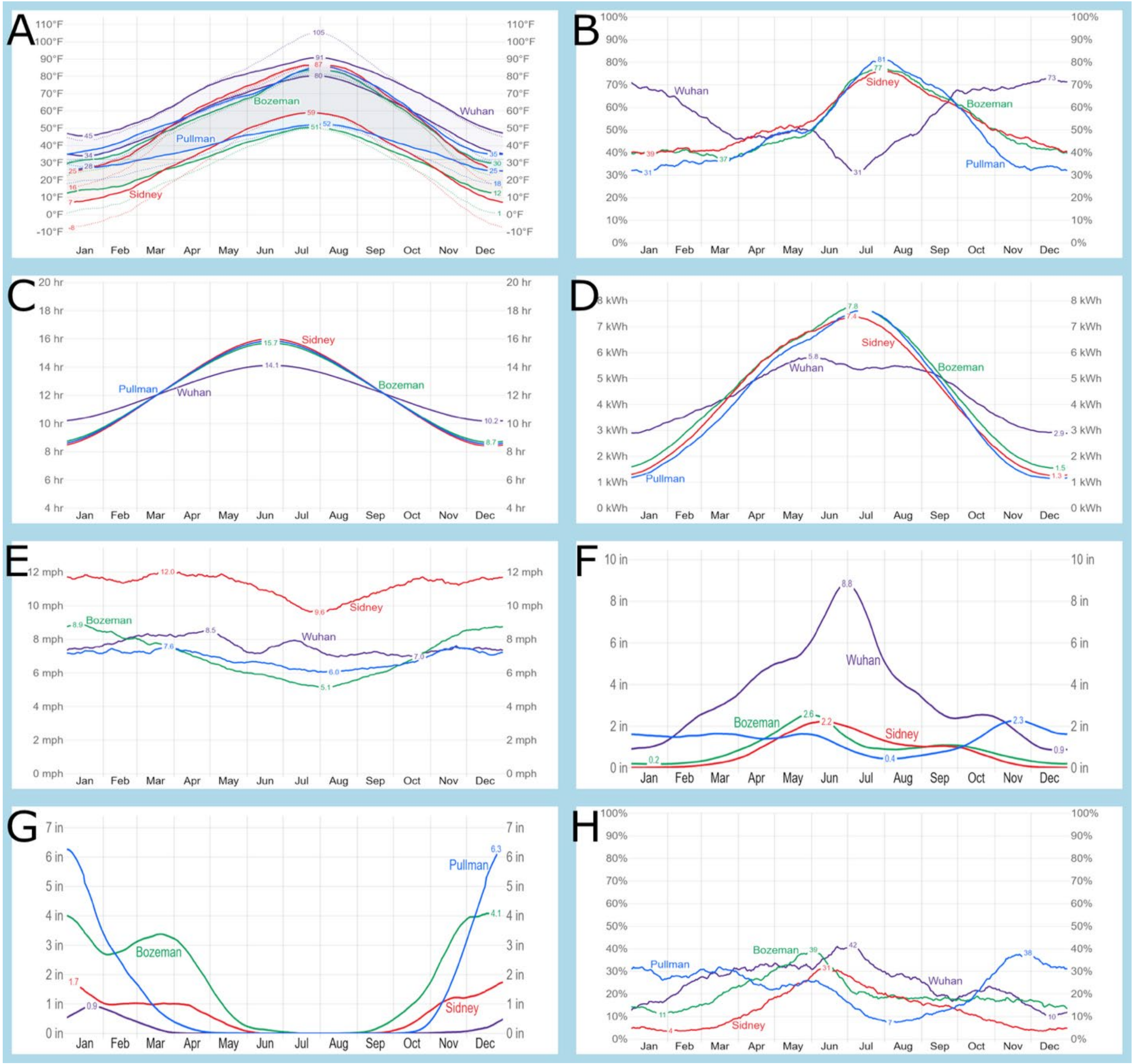

Supplemental Figure S1: Weather comparisons for the four growth locations during 2022. A: Average high and low temperature, taken daily at 2 meters above the ground. Solid lines are actual temperatures; thin dotted lines are corresponding perceived temperatures. **B:** Chance of clear skies, defined as the percentage of the time the sky is clear, mostly clear, or partly cloudy (less than 60% clouds). **C:** Hours of daylight, during which the sun is at least partly above the horizon. **D:** Average daily incident shortwave solar energy, measured per square meter. **E:** Average of mean hourly wind speed at 10 meters above the ground. **F:** Average monthly rainfall, taken over a sliding window of 31 days. **G:** Average monthly snow accumulation, taken over a sliding window of 31 days. **H:** Daily chance of precipitation, taken from the percentage of days in which precipitation is observed, excluding trace amounts. Data and graphs provided by weatherspark.com

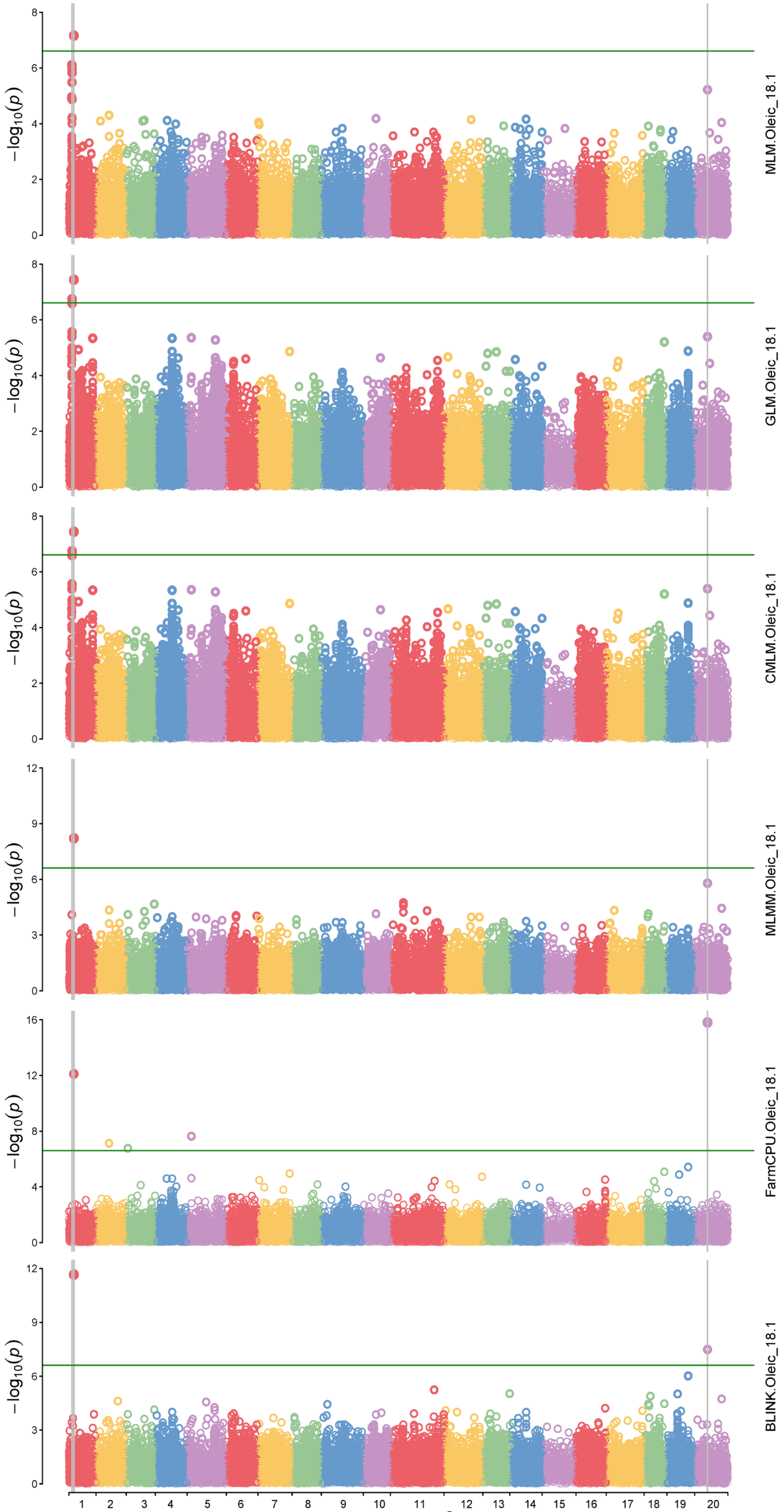

Supplemental Fig S2: Stacked Manhattan plots of the results of different GWAS models using the SNP marker set. The trait is oleic acid content taken from the Bozeman data. The solid green line is the significance level, based on the Bonferroni corrected threshold: -log10(0.05/#of tested SNPs). The dashed vertical line shows the consistent grouping of markers across the tested models. The chromosome number is on the x axis.

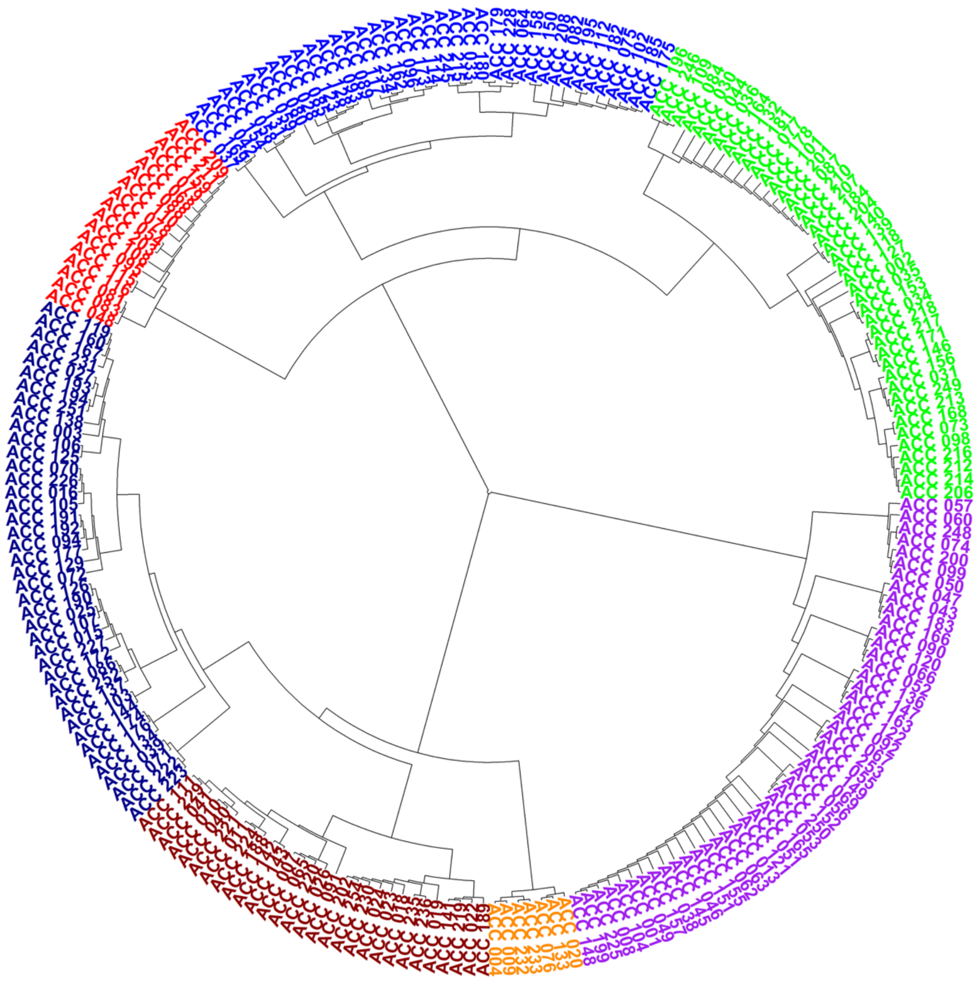

Supplementary Fig. S3. Maximum likelihood tree of *Cameina sativa* diversity panel.

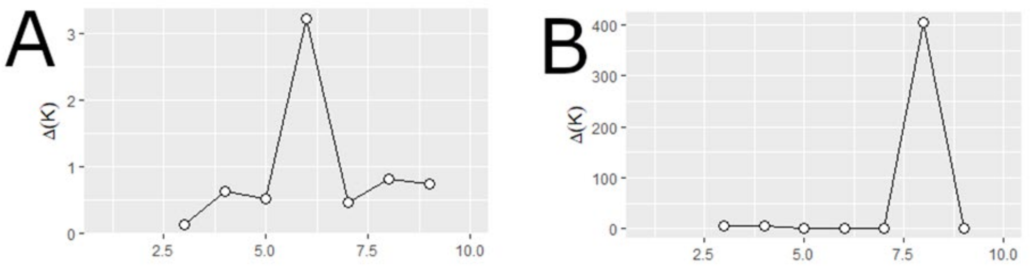

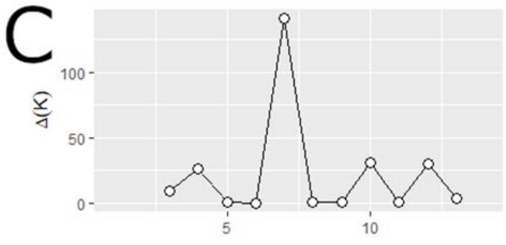

Supplementary Fig. S4. Evanno graphs of STRUCTURE results of the diversity panel. The X axis is the selected k value for the STRUCTURE test. **A:** Graph of the results for STRUCTURE run with no admixture and no population priors. **B:** Graph of the results for STRUCTURE run with admixture and non population priors. **C:** Graph of the STRUCTURE results with admixture and population priors.

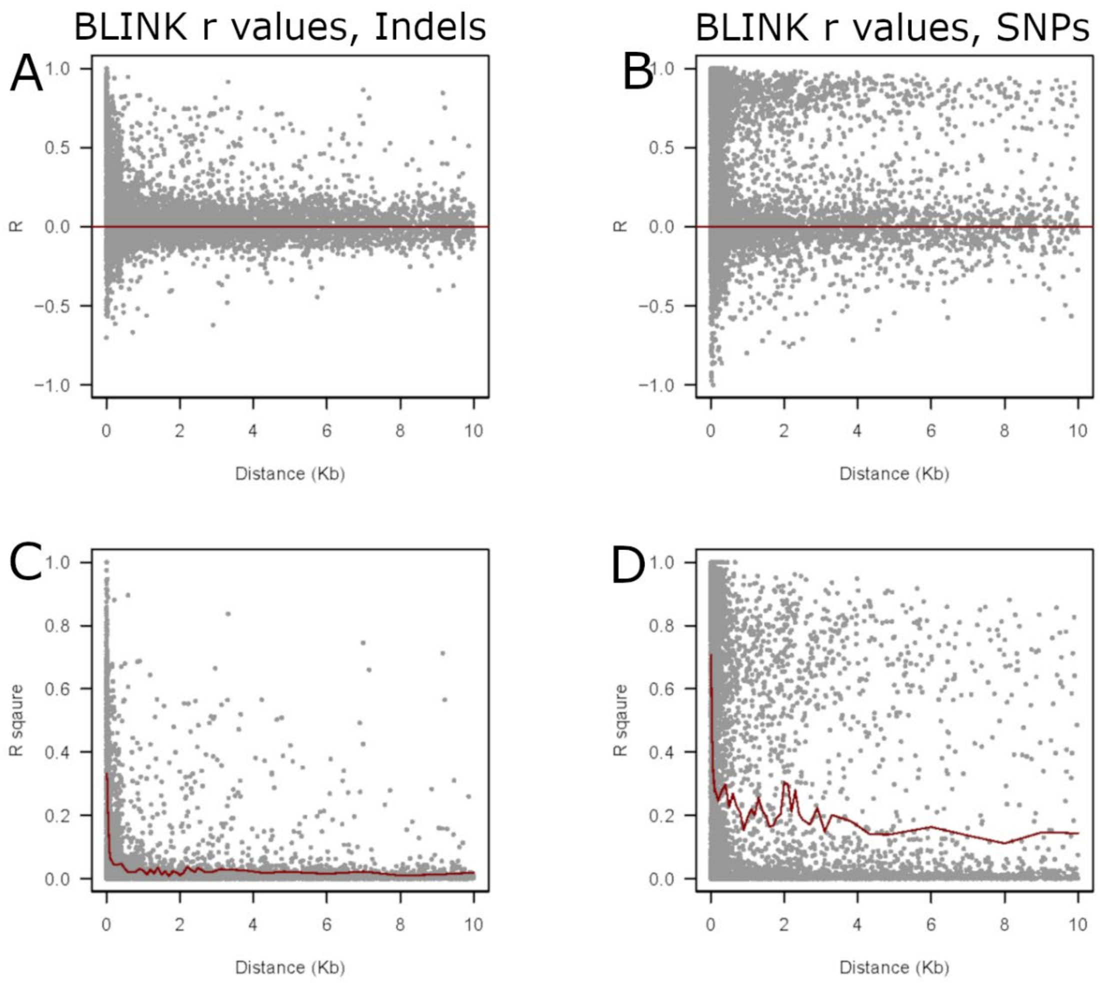

Supplementary Fig. S5. Linkage decay calculations by BLINK for both the SNP and indel markers. **A, C:** R values and R squared values from indel marker set. **B, D:** R values and R squared values from SNP marker set.

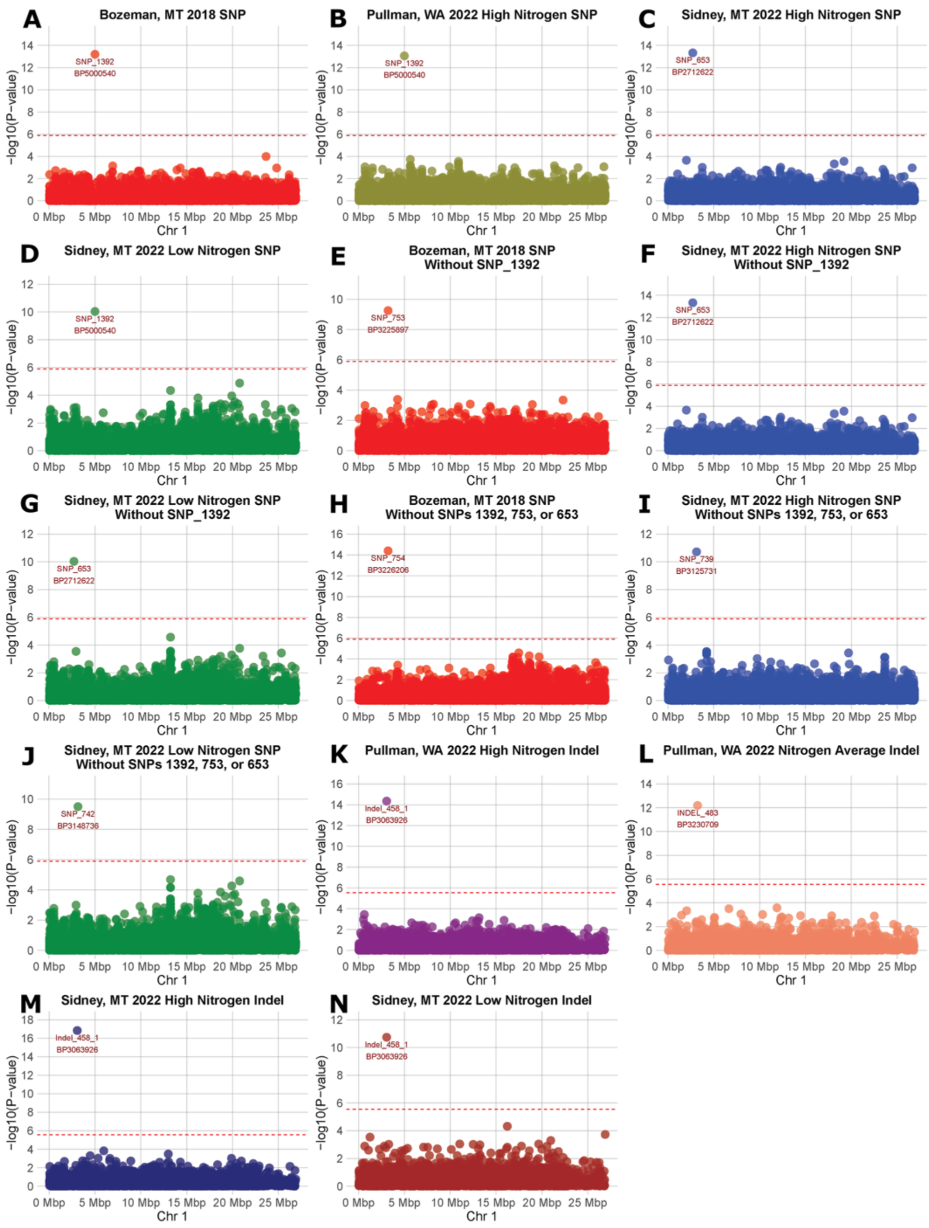

Supplementary Fig. S6: Determination of markers linked to 18:1 content by sequentially removing significant ones. The first pass of the GWAS identified SNP_1392, SNP_653 **(A-D**), and indel markers indel_458_1 and 483 (**K-N**). **E-G**: Removing both SNP_1392 and 653 revealed one more marker, SNP_753. **H-J**: Removing SNP_653 and 753 revealed SNP_754, 742, and 739. When indel_458_1, 483 and SNPs 754, 739, and 742 are removed, no additional markers are discovered on chromosome 1 associated with oleic acid.

Supplemental Table S1. Markers associated with increased oleic acid presence/absence compared to subpopulation identity. The 0, 1, and 2 for the marker columns show how many alleles that accession contains that are different from the reference. The values in the Group columns are the percent identity that accession has with the subpopulation in question.

ACC ID	SNP 653	indel 458_1	SNP 739	SNP 742	SNP 753	SNP 754	SNP 1392	Group 1	Group 2	Group 3	Group 4	Group 5	Group 6	Group 7
ACC_031	1	2	1	1	2	2	1	0.00%	0.00%	28.57%	14.29%	14.29%	14.29%	28.57%
ACC_035	1	1	1	1	1	1	1	0.00%	14.29%	28.57%	14.29%	14.29%	14.29%	14.29%
ACC_048	1	2	1	1	1	1	1	0.00%	28.57%	14.29%	0.00%	42.86%	0.00%	14.29%
ACC_050	1	2	2	1	2	2	2	57.14%	14.29%	0.00%	0.00%	14.29%	0.00%	14.29%
ACC_056	1	2	1	1	1	2	1	0.00%	14.29%	0.00%	0.00%	28.57%	42.86%	14.29%
ACC_059	1	2	0	1	1	1	0	0.00%	42.86%	14.29%	14.29%	14.29%	14.29%	0.00%
ACC_101	2	2	2	1	1	2	2	57.14%	0.00%	14.29%	14.29%	0.00%	14.29%	0.00%
ACC_114	1	2	1	1	2	2	1	28.57%	0.00%	28.57%	14.29%	14.29%	14.29%	0.00%
ACC_118	1	2	1	1	1	1	1	0.00%	0.00%	28.57%	42.86%	14.29%	14.29%	0.00%
ACC_133	1	1	1	1	1	1	1	0.00%	14.29%	42.86%	0.00%	28.57%	0.00%	14.29%
ACC_146	1	1	1	1	2	1	1	0.00%	0.00%	14.29%	14.29%	49.47%	14.29%	7.67%
ACC_149	1	2	1	1	1	1	0	0.00%	0.00%	14.29%	14.29%	14.29%	42.86%	14.29%
ACC_150	1	2	1	1	1	1	1	0.00%	0.01%	28.56%	14.29%	28.57%	14.29%	14.29%
ACC_153	1	1	1	1	1	1	1	0.00%	0.00%	15.76%	28.57%	14.29%	27.10%	14.29%
ACC_156	1	1	1	1	1	1	1	0.00%	0.00%	28.57%	16.51%	28.57%	14.29%	12.06%
ACC_158	1	1	1	1	1	1	1	0.00%	0.00%	18.84%	9.73%	28.57%	28.57%	14.29%
ACC_168	1	1	1	1	1	1	1	0.00%	0.00%	28.57%	14.29%	32.39%	14.29%	10.47%
ACC_171	1	2	1	1	1	1	1	0.00%	0.00%	28.57%	14.29%	28.57%	14.29%	14.29%
ACC_179	2	1	1	0	1	1	0	85.71%	0.00%	0.00%	0.00%	14.29%	0.00%	0.00%
ACC_181	1	2	1	1	2	1	1	0.00%	0.00%	36.14%	0.00%	57.14%	6.71%	0.00%
ACC_204	1	1	1	1	1	2	2	14.29%	0.00%	0.00%	14.29%	28.57%	28.57%	14.29%
ACC_206	1	1	1	1	1	1	1	27.63%	0.00%	28.57%	14.29%	14.29%	14.29%	0.94%
ACC_207	1	2	1	1	1	1	1	85.71%	0.00%	14.29%	0.00%	0.00%	0.00%	0.00%
ACC_212	1	1	1	1	1	1	2	0.00%	14.29%	28.57%	14.29%	14.29%	14.29%	14.29%
ACC_214	1	1	1	2	1	1	1	0.00%	0.00%	28.57%	28.57%	14.29%	14.29%	14.29%
ACC_216	1	1	1	1	1	1	1	0.00%	14.29%	28.57%	14.29%	14.29%	14.29%	14.29%
ACC_217	1	1	1	1	2	2	1	0.00%	9.83%	28.57%	14.29%	18.74%	14.29%	14.29%
ACC_218	1	1	1	1	1	1	1	21.50%	0.00%	28.57%	14.29%	14.29%	14.29%	7.07%

Supplemental Data 1: Camelina seed oil content and fatty acid composition across growth environments.
Click the icon to open the file.
